# ‘I Am No Longer Anxious When I Speak’: Experiences of People with Primary Progressive Aphasia Taking Part in a Biographic-Narrative Therapy (*Cope PPA*)

**DOI:** 10.3390/brainsci16020233

**Published:** 2026-02-16

**Authors:** Mirjam Gauch, Anna-Lena Köb, Julia Tanase, Julia Feldmann, Johanna Jochmann, Katharina Geschke, Helen Klaus, Oliver Tüscher, Isabel Heinrich, Sabine Corsten

**Affiliations:** 1 Department of Psychiatry and Psychotherapy, University Medical Center of the Johannes Gutenberg University Mainz, 55131 Mainz, Germany; migauch@uni-mainz.de (M.G.); anna-lena.koeb@unimedizin-mainz.de (A.-L.K.); julia.feldmann@unimedizin-mainz.de (J.F.); johanna.jochmann@unimedizin-mainz.de (J.J.); kgeschke@uni-mainz.de (K.G.); helen.klaus@unimedizin-mainz.de (H.K.); oliver.tuescher@uk-halle.de (O.T.); isabel.heinrich@unimedizin-mainz.de (I.H.); 2 School of Allied Health, Human Services & Sport, La Trobe University, Melbourne 3086, Australia; j.tanase@latrobe.edu.au; 3 Faculty of Healthcare and Nursing, Catholic University of Applied Sciences Mainz, 55122 Mainz, Germany; 4 Department of Psychiatry, Psychotherapy and Psychosomatic Medicine, University Medicine Halle (Saale) of the Martin Luther University Halle-Wittenberg, 06112 Halle, Germany

**Keywords:** primary progressive aphasia, biographic-narrative approach, reminiscence, life review, life-review therapy, quality of life

## Abstract

Background: Due to communication problems, people with primary progressive aphasia (PwPPA) are often affected in their self-image and experience a reduced quality of life (QoL). Biographic-narrative therapy is an effective approach to improve QoL in post-stroke aphasia. This study describes how PwPPA experienced their participation in the biographic-narrative intervention called *Cope PPA*. Methods: The intervention comprised a combination of five individual and seven group therapy sessions as well as the use of music and art therapy elements. Inclusion criteria were a capacity to give consent and sufficient visual/auditory abilities of PwPPA. Exclusion criteria were the presence of severe depression (MADRS > 35) or severe cognitive deficits (MMST < 10). After the therapy, PwPPA and their family members took part in half-hour semi-structured interviews. Interviews were analysed according to the reflexive thematic analysis by Braun and Clarke. Results: The qualitative analysis was based on a data set of 34 interviews. A total of six themes were identified: (1) Participation required adherence; (2) Materials were considered remarkable; (3) Storytelling was conducted in an aphasia-free area; (4) Group participation created a sense of belonging; (5) Experiences encouraged self-reflection and (6) Coping is lengthy and ongoing. Conclusions: The findings of our reflexive thematic analysis suggest that PwPPA experienced the intervention as meaningful. Some PwPPA described the effects of our intervention on their self-image. Others emphasised that coping with their condition was an ongoing process requiring continuous support.

## 1. Introduction

Primary progressive aphasia (PPA) is a neurodegenerative syndrome with a prominent communication disorder that is associated with Alzheimer’s disease (AD) or frontotemporal lobar degeneration (FTLD) [1]. A recent review and meta-analysis found that the pooled incidence of PPA was 0.52 per 100,000 people per year, with a prevalence of 3.67 [2], thus classifying PPA as a rare condition. With the progression of the underlying pathologies (AD or FTLD) people with PPA (PwPPA) experience additional cognitive and behavioural symptoms [3,4]. According to the consensus criteria developed by Gorno-Tempini et al. (2011, [5]), the PPA syndrome can be categorised into three variants: nonfluent (nfvPPA), semantic (svPPA) and logopenic (lvPPA). People with nfvPPA present with an agrammatic or effortful speech, people with svPPA show impaired naming and comprehension skills, while people with lvPPA primarily have difficulties in word-retrieval and sentence repetition [5]. Furthermore, variations in the typical variants are recognised (mixed PPA, [6]). As there is still no curative treatment for PPA, speech and language therapy is one of the most effective ways of helping people with this condition [7]. Several studies indicate that speech and language therapy can help maintain word retrieval performances over a longer period of time [8] or contribute to an improved fluency of speech [9].

PwPPA typically experience the first symptoms before the age of 65 [1] when they are still employed and would usually fulfil a whole bunch of social roles [10]. Diagnosis of PPA is often associated with a timely delay of 3 to 4 years [11,12] and accompanied by feelings of shock, anger, and hopelessness [13]. Adapting to communication difficulties, dealing with increased dependency, and profound relational changes are only examples of the challenges that PwPPA may face [13,14,15]. During the course of the disease, more than one-third of PwPPA develop depression, and up to half experience symptoms of anxiety at some point [16,17]. While research on post-stroke depression and interventions aiming to enhance the quality of life (QoL) of people with post-stroke aphasia increased in recent years, little is known about the concept of QoL in PwPPA [16].

In research, the picture of aphasia as an “identity theft” is widely spread [18]. Qualitative research on disease-related experiences of PwPPA speaks of an “multifaceted grief” that can be divided into three phases (before, during and after diagnosis) and encompasses affective, behavioural and cognitive symptoms [13]. Both metaphors, the “identity theft” and the “multifaceted grief”, suggest a strong subjective perception of loss that is irreversible and life-changing [14,19]. Although research on experiences of PwPPA and their family members is still limited [20], current findings show that it is necessary to address issues such as QoL, supporting PwPPA in coping with a changing self-perception, and facilitating a successful transition throughout the course of the disease [13,14].

A recent scoping review by Brinkman et al. (2024, [19]) aggregates findings of identity work among people with post-stroke aphasia and defines identity as a social construct negotiated through storytelling, influenced by social and personal factors. The authors explain the results of their review in a ‘Narrative Identity Model’ [19]. Of the 20 studies included, the biographic-narrative approach *narraktiv* was the only approach in which QoL was the measured and primary outcome [21]. *Narraktiv* is described as a structured yet non-directive approach. In this approach, the therapist primarily assumes a listening role, supports communication, asks resource-oriented questions, and provides space and time for self-thematisation. A mixed-methods analysis examined the positive effects of the *narraktiv* intervention, such as the significant improvement in QoL and mood, as well as the valuable experiences identified through qualitative interviews [21].

The five individual and seven group therapy sessions of *narraktiv* were carried out by a speech and language therapist (SLT) and an adult education professional who was specialised in biographical methods. During the individual sessions, participants were asked to tell their life story without interruption (“main narrative”), and then the therapist started asking subsequent questions, which were meant to reveal more details about the life story but also about the resources of participants. People with post-stroke aphasia experienced successful communication, which boosted their self-confidence in terms of their own narrative competence. During the group sessions, participants shared their stories in front of other people with aphasia and discussed personally relevant topics. Corsten et al. (2015, [21]) describe how people with aphasia valued meeting others with the same difficulties. In both—individual and group—settings, visualisation tools (e.g., timelines, pictograms, photographs) supported people with aphasia in expressing themselves. Both reflecting on the life story and sharing the life narrative lead to a more positive self-image and an improvement in QoL.

The qualitative interviews with participants in the *narraktiv* study revealed that people with post-stroke aphasia experienced positive changes in their ‘disease concept’ and feelings of ‘control’ [21]. The intervention further strengthened their sense of ‘agency’ and competence of ‘doing things’. These concepts are connected with the sub-themes of the ‘Narrative Identity Model’ of Brinkman et al. (2024, [19]), explaining how identity, if facilitated effectively, can enhance psychological well-being [19,22]. In consideration of these mechanisms of action, *narraktiv* as an approach [19] and participation in aphasia groups [23] has been demonstrated to have a positive effect on the experiences of people with communication disorders.

In PPA-research, impairment-based interventions are still dominating [7]. Nevertheless, there are a few studies describing QoL-enhancing approaches for the target group [24]. Although life story interventions have been demonstrated to enhance QoL in post-stroke and dementia conditions [21,25], there remains a paucity of evidence that is specific to the target group of PwPPA [14]. One of the few papers describing the effects of life story interventions on PwPPA is the work of Kindell and colleagues (2019, [26]). In their cross-case analysis with five individuals with svPPA and their partners, the contribution of life story work to the couple’s interaction is investigated, and an enhancement in interactional and emotional connections is described. Kindell et al. (2019, [26]) highlight the fact that life story work allows a focus on identity and has the potential to foster social relationships and well-being [26]. So far, no attempts have been made to transfer life story work to other variants of PPA or to investigate a larger sample.

Given the promising effects of *narraktiv* on people with post-stroke aphasia and the lack of approaches focussing self-image and QoL in PwPPA, we transferred the biographic-narrative therapy to the target group of PwPPA. In our project called *Cope PPA*, we first adapted the biographic-narrative approach *narraktiv* to the needs of PwPPA (‘Phase 1’) and then further evaluated our *Cope PPA* manual in a randomised controlled trial (‘Phase 2’). This article presents qualitative results from ‘Phase 2’ of *Cope PPA*, aiming to answer the following questions:

1. How did PwPPA experience their participation in the biographic-narrative intervention (*Cope PPA*)?
2. Which effects of the intervention have been observed by family members?
3. Which indications can be drawn from the qualitative results regarding underlying mechanisms of actions?

## 2. Materials and Methods

The *Cope PPA* study is a monocentric clinical trial with an adaptive design, allowing for adjustments to the manual according to the needs of PwPPA identified during ‘Phase 1’ [27]. The subsequent evaluation of the manual (‘Phase 2’) is conducted using a randomised controlled design involving a waiting period of ten weeks before receiving the same therapy. Despite the utilisation of a mixed-methods approach in the evaluation of the intervention, qualitative and quantitative data will be disseminated independently, ensuring that both approaches receive the requisite consideration. In this paper, we focus on the qualitative data that were collected during 34 half-hourly semi-structured interviews with PwPPA who participated in our clinical trial, as well as with one of their family members. The methods of ‘Phase 2’ of our study follow the Standards for Reporting Qualitative Research Checklist (SRQR, [28]), which can be found in Table S1. The study complies with the General Data Protection Regulations and was conducted in accordance with the Declaration of Helsinki. It was approved by the Ethics Committee of the Federal Medical Association of Rhineland-Palatinate (2023-17294).

### 2.1. Researcher’s Characteristics

The *Cope PPA* study was conducted by a research team of the outpatient memory clinic (GERMAN: Neuropsychiatrische Ambulanz mit Gedächtnissprechstunde) at the Department of Psychiatry and Psychotherapy of the University Medical Center (Mainz, Germany). The study design was conceived under the guidance of K.G., I.H. and S.C. The initial consultations were conducted by the study doctor (I.H.) and an SLT (M.G.). All therapy sessions were conducted by the same two SLTs (M.G. and A.-L.K.). Some of the participants already knew M.G. and/or A.-L.K. from the context of speech and language examinations, through previous group therapy programs or from the previous ‘Phase 1’. While M.G. was responsible for individual sessions, the group sessions were conducted jointly by M.G. and A.-L.K. The quantitative data was collected by M.G. with the support of J.F. The interviews conducted after the study, which form the basis of this paper, were collected and transcribed by three independent student SLTs (J.J., J.F. and H.K.). M.G. and four independent student SLTs helped with transcription or reviewed the transcripts. M.G. conducted the analysis, together with J.T., an SLT familiar with the used data analysis method. The final themes were discussed in a colloquium of independent SLTs with experience in aphasia therapy.

### 2.2. Inclusion and Exclusion Criteria

Participants were recruited by the outpatient memory clinic or cooperating outpatient speech and language practices. All recruited PwPPA had their diagnosis confirmed by blood biomarkers, in accordance with current guidelines [29]. While patients from the outpatient memory clinic were contacted via phone call, interested PwPPA who were not yet registered in our clinic were able to contact the research team independently. A recruitment flyer about the study was therefore created in simple language to inform PwPPA. Subsequently, interested PwPPA received detailed explanatory material and a consent form by letter or during their doctor’s appointment. The inclusion criteria were: firstly, a diagnosis of PPA based on the consensus criteria of Gorno-Tempini et al. (2011, [5]); secondly the ability to give informed consent; thirdly, sufficient visual and auditory capabilities. All PPA variants were included, as the *narraktiv* study demonstrated effectiveness across different aphasia profiles. The exclusion criteria were: firstly, pronounced cognitive deficits, defined with a cut-off value set at <10/30 points in the Mini-Mental State Examination (MMSE, [30]) and secondly, severe depressive symptoms, defined with a cut-off value of >35 in the Montgomery–Åsberg-Depression Rating Scale (MADRS, [31]).

### 2.3. Informed Consent

The ability to give informed consent was assessed by the study doctor (I.H.) during a preliminary interview. The assessment was based on the following criteria from international and national guidelines: (1) understanding relevant information, (2) understanding the implications for one’s own situation, (3) reasoning about the information, and (4) expressing a treatment choice [32,33,34]. During the preliminary interview, interested PwPPA were informed about the programme’s voluntary nature and its independence from other therapies. PwPPA were informed about the advantages (e.g., possible improvements in QoL, positive experiences of group therapy), disadvantages (e.g., time/transportation commitment) and risks (e.g., stress related to meeting other participants). The attention of interested PwPPA was drawn to the passage in the consent form that mentions the video recording and the associated data protection principles of the study. There was also room for further questions.

### 2.4. Clinical Trial Design

**Figure 1:**
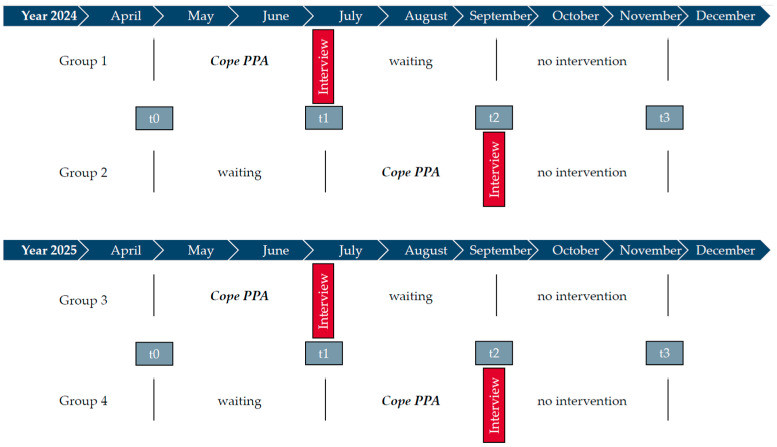
Timeline of the *Cope PPA* intervention with two cohorts (group 1 and 2 in 2024; group 3 and 4 in 2025): Colours of the figure are red (semi-structured interviews after the *Cope PPA* intervention), grey (quantitative testing) and blue (years/months of our intervention). During the ten weeks of intervention, participants received either individual therapy sessions only (week 1), group therapy sessions only (weeks 4, 7 and 10), individual and group therapy sessions (weeks 2, 3, 6 and 9), or no therapy (weeks 5 and 8). During the therapy-free weeks, participants had time to reflect on the treatment and a therapist contacted them by telephone. Each individual or group therapy session lasted 90 min.

Considering the rarity of the disease, a more protracted recruitment period was deemed necessary, extending over a duration of 16 months (year 2024/2025). To avoid protracted waiting times, the intervention was divided into two cohorts. Following the recruitment phase, PwPPA participants were randomly allocated into therapy groups comprising a minimum of four and a maximum of six participants. The first group was randomly allocated to either the therapy or the waiting condition by means of a roll of a die. Depending on the result, the second group was assigned to the other condition. The initiation of the intervention by the cohort of PwPPA was contingent upon the attainment of a sufficient number of participants. Two groups (1 and 3) received therapy immediately after randomisation, two groups (2 and 4) were enrolled in the waiting condition and received the intervention with a delay of 10 weeks (Figure 1).

### 2.5. The Cope PPA Intervention

In their individual therapy sessions, PwPPA had time to tell their life story without interruptions. It was the job of the SLT to facilitate storytelling by providing cues or pictograms and by paraphrasing what was said. After a disclosure of their life narratives by PwPPA, the therapist employed a series of internal enquiries (e.g., ‘*You mentioned your childhood. What else comes to your mind about that time?*’) and external enquiries (e.g., ‘*Who or what makes you happy?*’) designed to elicit introspective reflection and to make self-theories and future-perspectives more explicit. Especially, the final therapy sessions were used to elicit subjective definitions or arguments of personal relevance (e.g., ‘*What does health mean to you?*’ or ‘*Has your understanding of health changed after the diagnosis?*’). During group therapy sessions PwPPA came together to discuss biographical and everyday topics (e.g., ‘Family/friends’ or ‘Health/disease’). Non-verbal and creative arts were used to support self-expression and thus facilitate identity work (e.g., painting or Lego Serious Play). The therapists encouraged conversation by using memory and communication aids and moderated group discussions to ensure that everyone had the opportunity to speak. An overview of the *Cope PPA* manual is given in Table S2.

### 2.6. Data Collection

The testing (t0, t1, t2, t3) and the therapy sessions were video-recorded, semi-structured interviews were audio-recorded. Audios and videos were stored on the local servers of the Department of Psychiatry and Psychotherapy of the University Medical Center (Mainz, Germany). Interview transcripts were saved on the same servers. During the transcription process, all PwPPA were assigned a random identification number, and identifying information was anonymised. The quantitative evaluation at the three test time points included standardised, psychometric measurements of QoL, life satisfaction and depressive symptoms. Additionally, mood was measured before and after the first and fifth individual therapy sessions as well as before and after the first and seventh group therapy sessions. Quantitative findings of our intervention will be published elsewhere in the future. The qualitative data were collected through semi-structured interviews with PwPPA and their closest family member. The interview guideline aimed to capture the views and satisfaction with the intervention by asking questions such as: “*How did you find the individual and group therapy sessions?*”. The interviews also aimed to obtain feedback on specific methods (“*What do you think of drawing a lifeline?*”) and effects on an individual’s self-perception (“*Would you say you see yourself differently since participating in Cope PPA?*”). The questions asked of family members were similar to those asked of PwPPA but focused slightly more on the observable effects beyond the therapy room (“*When you look at this scale, what do you think your partner’s mood was like?*”). The complete interview guidelines for PwPPA and their family members can be found in Table S3. During the interviews, photos of the *Cope PPA* materials were presented to help participants remember specific materials and methods. Additionally, life story books and memory boxes were viewed with participants to observe their reactions and spontaneous interactions.

### 2.7. Data Analysis

**Figure 2:**
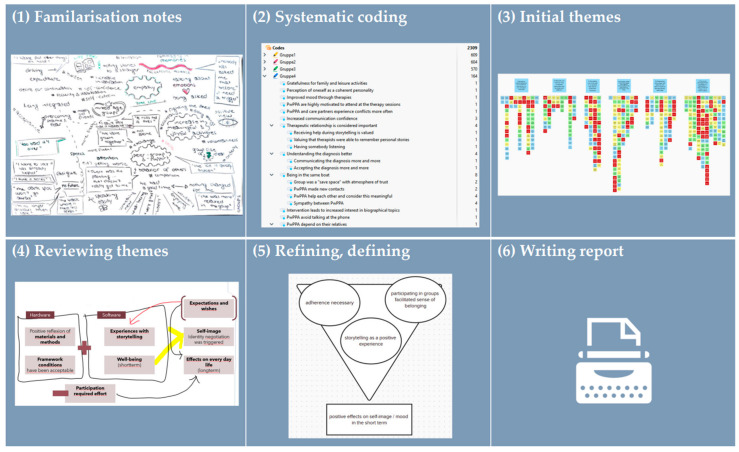
Procedure of reflexive thematic analysis according to Braun and Clarke (2022, [37]).

The audio-recorded interviews were transcribed orthographically by M.G., following the guidelines of Dresing and Pehl (2018, [35]). Due to the aim of our research, which was to explore the experiences of participants in the *Cope PPA* study, reflexive thematic analysis was chosen as an inductive method [36]. The data was analysed using a constructionist approach [37]. In line with this approach, particular attention was paid to the language used by PwPPA, based on the theory that ‘ways of talking create reality’ (p. 180, [37]). In addition, the analysis was carried out to ‘make sense’ from the data, as Braun and Clarke (2022) put it (p. 179, [37]). To ensure a structured analysis, the 15-point checklist of Braun and Clarke (2013, [38]) was used. The completed checklist can be found in Table S4. During step one of the analysis, M.G. familiarised herself with the data while taking notes by hand. Step two contained a systematic coding of statements relating to the research question using the MAXQDA software (VERBI Software, 2025, version 24.11.). Overlaps and smaller codes were combined. Samples from the available data were coded by independent colleagues in working groups during national methodology workshops or in an SLT colloquium. The selection was made by M.G. and was based on content (e.g., contrasting cases). During step three, initial themes were generated from collated data using a Mural board (https://app.mural.co/) (accessed on 25 September 2025). Step four, which contained the development and review of themes, was organised in a collaborative procedure between M.G. and J.T. Based on this collaborative process, M.G. revised the themes again in step five and presented them to the SLT colloquium, with the aim of supporting in-depth reflection and testing the comprehensibility of the themes in an uninvolved peer group. The themes were adjusted again based on collective knowledge, after which M.G. wrote the data analysis report in step six, which was then critically reviewed by all co-authors. This report now forms part of the subsequent Section 3. Due to the study design (Figure 1), the complete data analysis was performed in an iterative process. During the later stages of data collection, interviews increasingly confirmed and elaborated existing patterns rather than extended the thematic structure of the analysis. The process of the data analysis is visualised in Figure 2.

## 3. Results

**Figure 3:**
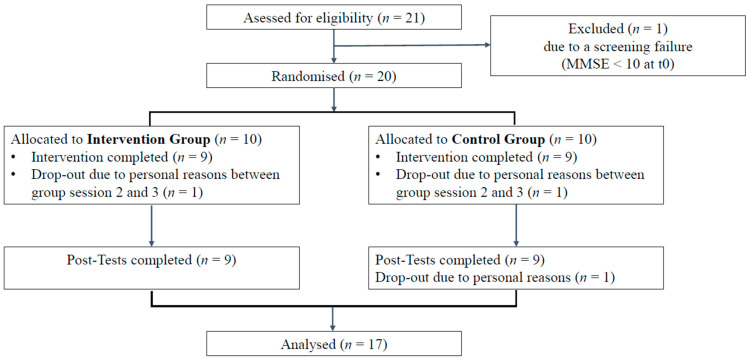
Flow chart for recruitment and study procedure.

Initially, a sample size of 24 was targeted. A total of 21 participants were successfully recruited for the study. However, one participant was excluded at t0 due to a screening failure (participant did not meet the inclusion criteria, MMSE > 10 at t0). Two other participants withdrew from the study due to personal reasons. P15 withdrew from group therapy because she did not feel comfortable in this setting. Instead, she decided to continue with individual therapy only. A complete flow chart for recruitment and study procedure can be found in Figure 3.

The demographic data of the seventeen participants included for analysis is presented in Table 1. In addition to PwPPA, 18 family members were also interviewed (in one interview, both the wife and daughter of a participant took part). The average age of the participants was 71.5 years (SD 10.4) with an average age at symptom onset of 66.0 years (SD 3.1) and an average age at PPA diagnosis of 69.8 years (SD 10.0). The gender ratio was reasonably balanced (47% female). All PPA variants were represented. The average score for cognitive abilities, as defined by the MMSE, was 21.0 (SD 5.7). The rate of successful communication, as measured by the Scenario Test (ST, [39,40]), was 88.5% for the present sample.

### Themes

**Figure 4:**
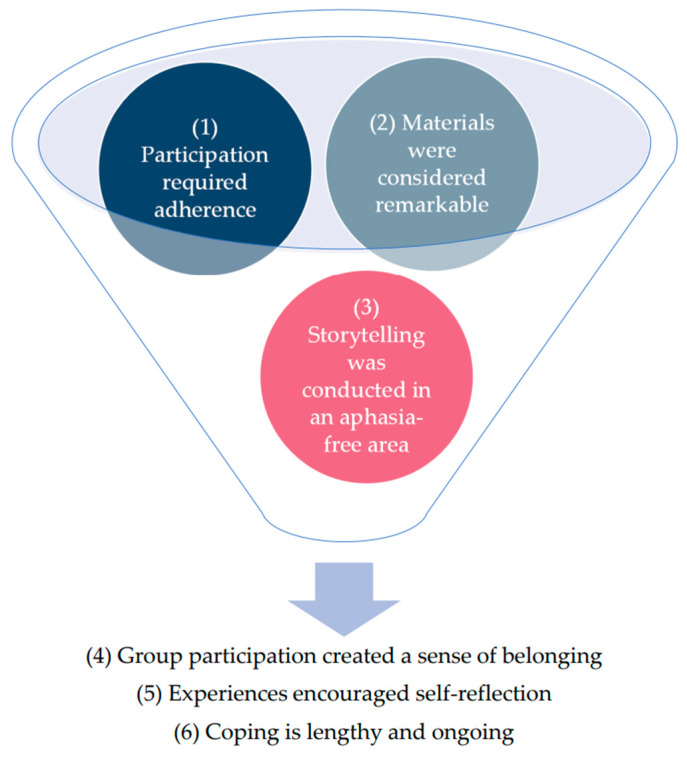
Visualisation of themes in a simplified form (designed by the author using PowerPoint images, Version 2401): While the first three themes are to be considered as influencing factors ‘within’ the *Cope PPA* intervention (symbolised by the area inside the funnel), the remaining themes focus more on the effects that have resulted ‘from’ participation (symbolised by what comes out of the funnel).

In total six themes were represented in the data: (1) Participation required adherence; (2) Materials were considered remarkable; (3) Storytelling was conducted in an aphasia-free area; (4) Group participation created a sense of belonging; (5) Experiences encouraged self-reflection; and (6) Coping is lengthy and ongoing. The link between the themes is shown in Figure 4. All themes and findings of the qualitative analysis are described below. Key quotes are embedded directly in the text. The quotes are from participants with PPA (P1–P24) or their family members (FM1–FM24) in groups 1–4.

**Theme 1: Participation required adherence—PwPPA and their families considered the intervention to be meaningful and therefore accepted associated efforts.**

PwPPA and their family members described framework conditions of *Cope PPA* as appropriate. They valued the structured procedure consisting of initially set therapy sessions within a limited period (“*That was really great. Very well organised, too.*”—FM13, group 1). The fact that the intervention was planned as a clinical trial made it particularly meaningful for PwPPA. Participants described how important it was to them to support research (FM24, group 2). The duration of therapy sessions was considered appropriate; only a few individuals stated that 90 min were too short. The frequency and intensity of the intervention were perceived differently among PwPPA. While some perceived the intervention as intense, others said that they would have liked a higher frequency. The group size of group 2 (*n* = 5) was described as ideal by P17, while P14 stated that the small size of group 3 (*n* = 3) suited him because of his tinnitus. Participation in the *Cope PPA* study was supported by various family members who provided transportation services. Especially those located at a geographical distance from the outpatient memory clinic stated they had little time for anything else besides their therapy sessions. In some cases, PwPPA travelled on their own (by foot, by car or by public transport), which reduced the burden on family members and gave spouses an extra time for themselves. Family members felt supported by the regular telephone calls in weeks when no therapy session was conducted, as the following quote illustrates:

*“Yes, I also thought it was nice that [therapist’s name] would call if there were no appointments for a week. Yes, you had the feeling that these were people who cared about my husband, who… Yes, how should I put it… Such interest… I thought it was nice, yes. And that someone was simply interested and cared. So that was a nice feeling. At least for me. (laughs)”*

FM14, group 4

**Theme 2: Materials were considered remarkable—the methods used encouraged self-reflection.**

Materials and methods of *Cope PPA* were widely valued, although some PwPPA expressed their reservations about the use of creative arts (“*Well, I’m not the man for that, uh, that’s not my thing.*”—P21, group 2). The participants found drawing their lifeline helpful in identifying and visualising major turning points in their lives. The use of Lego Serious Play as a method was perceived as enjoyable. PwPPA found the material easy to access and were proud of the “perfect world” that they created in a group effort (“*Yes. Isn’t the world beautiful?*”—P1, group 1). The variety of methods was valued by P2 (group 4) illustrated through quotes like the following:

*P2: “There were certain things that I hadn’t used before. Like when it was just about taking a picture or something like that, right? I found that very interesting. I had never done that before, and I enjoyed it.*

*I: “Okay, so generally, if I understand correctly, you liked the concept of the group with the others, but you also liked the content that was created in the group?”*

*P2: “Well, to sing. I haven’t sung in ages. I don’t think the others have either. And that was just very, very nice.”*

The life story books and memory boxes created in the individual therapy sessions were also used by participants outside of the program and promoted communication between spouses but were also a tool for identity work. PwPPA appreciated the therapist’s support in creating the albums and said that they would not have been able to do it on their own (“*And I thought it was really great with the pictures and everything we did there. And I thought it was really, really great. I don’t think I would have gotten that there, because I tried it.*”—P19, group 4). PwPPA were often asked to bring materials to the group therapy sessions (e.g., personal photographs or interesting newspaper articles). PwPPA took these requests very seriously and referred to them as their “*homework*” (P18, group 5). Family members reported that thinking about the topics for the next meetings would already improve the well-being of PwPPA, but that the work assignments (e.g., bringing in a newspaper article) could not be completed independently by PwPPA (FM18, group 5). The communication rules of the group sessions were considered helpful, although some participants regarded the rules as self-evident manners that did not need to be communicated. Feedback was provided on the comprehensibility of the rules and stated that certain rules (such as “Sending I-messages” and “Balancing listening and talking”) were not easy to understand by PwPPA.

**Theme 3: Storytelling was conducted in an aphasia-free area—PwPPA described the atmosphere created by therapists as trustworthy.**

PwPPA appreciated the atmosphere during therapy and attributed this to the relationship of trust they had built up with their therapists. Active listening and assisted communication created an accessible environment in which storytelling was perceived as easy. The following quote mirrors the experience of the phenomenon described in Theme 3 as an aphasia-free area:

*“As soon as I get here to the parking slot and go in here, I hardly start stuttering. When I’m somewhere else, it’s there/it’s stronger.”*

P17, group 2

The interaction during the sessions was rated as positive, and the depth of the discussions was appreciated, as the following quote illustrates:

*“(…) I had a few episodes where I said, oh yes, back then I did this and that. And then she (the therapist) asked, “Yes, what was that?” And so on. And that was/I thought that was quite good, because when I talk to someone else somewhere else, that’s how it is. It’s a bit more superficial. And THAT was actually good, yes. To follow up on that. How I was doing and how I had changed. From youth to today, you change, even without illness (laughs). Yes. And I thought that was good. And I also learned something about myself.”*

P17, group 2

Occasionally, PwPPA expressed that they preferred not to disclose too much information during individual therapy sessions where there was a lot of focus on them (P21, group 2). The therapeutic relationship was described as positive. The endeavours of the therapists (“*she made a tremendous effort, and her willingness to help was enormous.*”—P9, group 1) and their ability to put themselves in another’s place (“*she is so empathetic.*”—P22, group 2) were valued by PwPPA.

**Theme 4: Group participation created a sense of belonging—being part of the group increased well-being in the short term.**

PwPPA described a sense of belonging and described their feelings with various metaphors (“*sitting in the same boat*”—P2, group 3/“*We were a family*”—P19, group 4/“*It was like a circle, uh, where you felt comfortable*”—P21, group 2). The positive atmosphere influenced how PwPPA felt during the group therapy sessions (“*Light. Lively*”—P9, group 1). Improvements in mood were also evident immediately after the sessions, as the following quote illustrates:

*“When I went back, I mostly walked, and there it was, I had a completely different, much better feeling. A physical feeling. And overall. So, I always walked the whole way, and I was always really happy. Yes.”*

P2, group 3

Many comments indicated that PwPPA compared themselves to each other. This predominantly had positive effects, as PwPPA enhanced their self-esteem by comparing themselves to participants who were worse off or by finding their place in a group where everyone could contribute their strengths and weaknesses. However, some participants found that meeting other PwPPA was confrontational and made them aware of the possible effects of the disease:

*“It just made me realise that it would be unpleasant because we had such difficult cases in the group. I think when it comes to that, I’ll hide away somewhere in a retirement home.”*

P5, group 1

Not all PwPPA were present at all meetings due to holiday plans that were not entirely compatible with the study appointments, or because they no longer wanted to participate in group therapy (P15, group 4). Some participants who attended all therapy sessions regretted the absence of their peers. Occasionally, concerns were raised about members staying away. This highlights the sense of community that developed among the group over time.

**Theme 5: Experiences encouraged self-reflection—lasting effects on the self-image have been described.**

Beside short-term improvements in mood which are described in Theme 4, there have also been lasting effects for some PwPPA. For some PwPPA the intervention further led to an increase in contacts (“*[name of P11] called me, also by phone, and I have a very good relationship with her.*”—P9, group 1). Family members described how PwPPA showed an increased desire for socialising and for sharing personally relevant topics. This was observed within the context of family life and social circles and was directly related to the self-reflection stimulated by storytelling. Family members reported that they were able to experience their loved ones more like they used to be and that the study had led to an increase in days with a reduced impairment (“*And now there’s this movement where I sometimes feel like the old (name of P2) is coming back, who has these moments of clarity.*”—FM2, group 3). Family members described an increase in self-care practice and intensified interest in activities of their loved ones (“*I do think that he is now doing more things that he enjoys. (…) He has been spending a lot of time with his music again*.”—FM17, group 2). The interviews revealed that some PwPPA developed awareness of their own limitations and increasingly accepted them. In some cases, the therapy helped PwPPA to communicate their diagnosis more openly. The self-perception of participants varied within the cohort. Some PwPPA felt their self-image remained stable (“*still a bit crazy, but still in a good mood.*”—P4, group 3); others reported seeing themselves differently since participation (“*I am no longer anxious when I speak.*”—P22, group 2/“*I’m already feeling a bit better, (…) more stable sounds stupid, but a bit stronger, a bit firmer*.”—P7, group 1/“*I felt a little bit taller.*”—P2, group 3).

**Theme 6: Coping is lengthy and ongoing—PwPPA and their families wish to receive continuous support.**

PwPPA across all groups asked for a continuation of the intervention and expressed regret about the conclusion of the therapy sessions. One participant (P2) suggested continuing the intervention online in the future to reduce travel-related burden. The fact that PwPPA continued to describe their life with PPA from a negative perspective and continued to express feelings of frustration about their symptoms shows that the process of accepting the illness is ongoing. Although a few participants reported an increase in communication confidence (see Theme 5), others described their struggles to feel self-confident despite their speech disorder, as the following excerpt from the interview with P11 (group 1) illustrates:

*I: “(…) what’s stopping you from daring to speak up?”*

*P11: “That I stutter and don’t know what to say. Yes, word-finding difficulties and, um, then you feel so helpless and ashamed and, well… it’s… and, yes…”*

*I: “And these feelings you’re describing, haven’t they changed because of the study?”*

*P11: “It’s working inside me. I want to, yes.”*

PwPPA describe the intervention as a change and a contrast to their everyday life—a life, which is greatly affected by the disorder (“*That was just great, because we are all actually very sad when we take a close look at what is going on with us at the moment, and that was just wonderful (…) I think it benefited us all a lot.*”—P2, group 3). The family members described the intervention as the first step away from negative emotional and mood developments. However, this first step must be followed by further steps, as continuity of care is important to them. During the semi-structured interviews, some PwPPA indicated that they could no longer remember the content of group therapy sessions or who was present (P4, group 3). Family members further expressed concerns about how much their loved ones could remember or process the content of the intervention (“*He always remembers things like that, but sometimes you realise that nothing is getting through to him.*”—FM3, group 3). This also prompted family members to question the long-term effects of the therapy. Although many PwPPA considered storytelling “*easy*” (P18, group 4), others put themselves under pressure due to questions touching certain phases of their lives that they had fewer memories of (P4, group 2). PwPPA and their family members repeatedly described feelings such as grief and expressed the rays of hope they see in their everyday lives. Some family members expressed their desire and need to be more involved in therapy (FM18), while others expressed their burden of responsibility and wished to hand over responsibility to professional practitioners (FM19).

## 4. Discussion

The aim of this study was to find out how PwPPA perceived their participation in the *Cope PPA* project and what effects of the intervention had been observed by family members. Additionally, we tried to find out more about the mechanisms of action which might be relevant for identity work in PwPPA.

Reflexive thematic analysis revealed that, while participation was considered meaningful, it required commitment and effort from PwPPA and their family members, who had to attend regular in-person therapy sessions, often requiring long travel or cancelled appointments. Compared to other clinical trials enrolling PwPPA, the *Cope PPA* intervention can be considered moderately frequent. While speech-systematic approaches for PwPPA are already being provided with six therapy sessions per week [41], psychosocial approaches tend to be provided less frequently (e.g., bimonthly [42]). In a similar way does duration of therapy sessions vary between clinical trials: speech and language therapy interventions are generally delivered with a minimum of 25 min and a maximum of 120 min per session [7,43]. Occasionally, QoL-enhancing interventions like so-called “Aphasia camps” are held as a weekend event once a year [44]. The different perceptions of the included PwPPA and their family members suggest that the perceived intensity and willingness to make an effort depend on individual resources (e.g., do PwPPA travel independently/can family members take turns with the transport services) and character traits (e.g., are PwPPA optimistic/resilient). This assumption would be consistent with the findings of other studies that have discussed personality traits such as resilience or ambition/proactivity as decisive for outcomes such as QoL [44]. The rather small group size of our intervention groups was valued by PwPPA. Although a current review article [45] shows that some group interventions, especially those who are delivered online, include much more participants (up to 30 PwPPA and caregivers).

Materials and methods of *Cope PPA* were considered remarkable as they were new for PwPPA. The feedback of participants and their reactions on photographs presented during the interview showed how especially creative art methods facilitated self-reflection. Current systematic reviews have shown the potential of art therapies in dementia care facilitating in-the-moment interaction and communication [46]. The use of creative art therapy for PwPPA has only been described in a few studies so far [42]. While general reports on alternative communication in acquired language disorders more frequently refer to drawing as a means of compensation [47], there are only very isolated references to its specific use for PwPPA [48]. Kindell et al. (2018, [49]) have already demonstrated how music can improve interaction between people with svPPA and their family members. Additionally, it is known that some PwPPA (primarily individuals with FTLD) can develop new musical or art skills over time [50,51]. Accordingly, the use of creative arts in therapy with PwPPA appears warranted. The life story products created in *Cope PPA* were valued by participants, consistent with an integrative review, which postulated that such products of life story work helped people with dementia to maintain their sense of identity [52]. The feedback on the communication rules showed that not all PwPPA had understood them and that they should be simplified further.

Due to supported conversation the biographic-narrative intervention *Cope PPA* was perceived as accessible for PwPPA. The interaction with therapists was described as consistently positive. Some PwPPA stated that communication in therapy was special and significantly differed from other conversations. Likely, this was also due to the depth of the questions and the degree of self-reflection achieved. The positive experience of self-reflection is one of the key mechanisms of biographic-narrative therapy. The fact that we repeatedly identified this in our data is consistent with the theory of Davies et al. (2024, [14]) that identity work is feasible and beneficial for PwPPA. The therapeutic relationship, which is currently receiving increased attention in aphasia therapy research, was also mentioned in the interviews by PwPPA and their family members. In an essay on psychotherapy for people with aphasia, two key factors that influence the therapeutic relationship and the outcome of treatment are mentioned: first, the working alliance, which is based on a consensus between patient and therapist and interpersonal bonds; second, a resource-oriented view that is reflected in the therapist’s attitude and her/his approach to therapy [53]. It is likely that these criteria also apply to the treatment of PwPPA and are particularly relevant in psychosocial interventions.

It was highlighted by PwPPA that being part of a group created a sense of belonging. The participants used metaphors to express the special intimacy within the groups. Overemphasising the choice of words used by PwPPA is not entirely unproblematic because of potential semantic paraphrases. Nevertheless, expressions such as “*sitting in the same boat*” or “*We were a family*” have a special symbolic power. From a constructionist perspective [37], the group feeling described above could be seen as a meaningful mechanism of action, which may have an empowering force for some PwPPA. At this point, however, it must be noted that negative feelings were also described in isolated cases, as group therapies also mean confrontation with the possible effects of the disease in later stages. One participant decided against continuing with group therapy because she did not feel comfortable. These observations underscore the findings of Volkmer et al. (2025) and suggest that grouping people according to their cognitive abilities, personality traits, or interests can be beneficial [45]. Outside of randomly assembled therapy groups, these aspects could be taken into consideration in the future. The sense of belonging among our participants was based on various aspects. For example, the experience of not being alone with an illness and mutual sympathy. PwPPA stated that they had learned from each other within the group, something that is a typical effect of support groups and was also described by participants in the *narraktiv* study [21]. PwPPA described how the group had taught them patience and how good it feels to help each other. This is remarkable because particularly PwPPA with underlying FTLD might experience apathy instead of strong feelings of companionship [54]. It is possible that our therapy had effects in the area of social competence, which we had not clearly investigated or intended. In relation to the sense of belonging, the short-term effects on psychological well-being and mood improvement were described. This is one of the most significant effects described by PwPPA and their relatives. We agree with Kindell (2015, [55]) that such in-the-moment effects are highly relevant, and assume that short-term improvements in mood are sometimes more realistic than other long-term effects.

Nevertheless, some participants also described how their participation led to long-term improvements in self-image. These observations are consistent with the mechanisms of action expected by the research team from biographic-narrative therapy, as they correspond to the findings of Corsten et al. (2015, [21]). One such mechanism is ‘doing things’, a concept reflected in our data: PwPPA showed greater interest in their environment and pursued their interests more intensively as a result of the intervention.

PwPPA and their family members who did not describe any long-term improvements in their self-image repeatedly talked about experiences of grief. This grief would dominate their everyday life and would not simply disappear as a result of a study, without a curative claim. The interview data showed that not everyone’s primary desire is for psychosocial therapy; rather, there is a great need for disease-modifying therapies. At a time when promising approaches are being developed, the fluctuation between hope and despair is a key challenge. While some parallels can be drawn between Theme 5 and the findings of the *narraktiv* study, Theme 6 represents something new that may be typical of PwPPA. While people with post-stroke aphasia experienced a sense of ‘control’ and were motivated by social upward comparison to improve their aphasia rehabilitation, PwPPA and their family members referred to the inevitability of progression. This might be interpreted as a subjective loss of control, caused by the lack of curative therapies to this day.

### Limitations

Our analysis yielded several findings and revealed some methodological limitations. Notably, the same researcher (M.G.) was responsible for conducting the therapies and analysing the qualitative data. This dual role arose for pragmatic reasons, as the study formed part of a doctoral thesis and funding was only available for one researcher. Due to a lack of time and financial resources, independent coding could not be applied to all the data material. This carries the risk of bias, which could only be partially offset by techniques designed to increase credibility.

The study comprises a dataset that was limited from the outset by the design of the study. As PPA is a very heterogeneous syndrome, each additional participant could have contributed new experiences. Nevertheless, we consider our sample of 34 interviews with PwPPA and their family members to be sufficient to achieve a deep understanding and a certain level of information power [56,57].

Some participants knew the therapists (M.G. and A.-L.K.) from previous appointments at the memory clinic, so a therapeutic relationship already existed before the start of the study. These circumstances may have increased adherence to the intervention and contributed to effects such as social desirability in the interviews. To reduce the effects of social desirability bias in the interviews, only interviewers who were not involved in the therapies were selected. We considered the fact that the therapists M.G. and A.-L.K. were familiar to the participants to be a strength. As a long-term therapeutic relationship is certainly helpful in view of Theme 6, considering the expressed desire for continuity among PwPPA. Even though the *Cope PPA* intervention ended after ten weeks, contact with the therapists continued over a longer period of time and continues to this day.

It should further be noted that the therapy was provided in two cohorts, one year apart and that interview data was therefore collected iteratively (see Figure 1). These circumstances imply potential risks for both confirmation and maturation effects. Participants received individually tailored therapies and were often able to choose between different materials themselves (e.g., whether to create a memory box or a life story book, or which painting/drawing materials to use), which produced a very distinct experience for all participants. Nevertheless, all interviews were included in the analysis to provide insight into the collective experience of PwPPA within *Cope PPA*.

The perspective of family members who were not present during the therapy sessions but were asked to describe the effects secondhand as close observers was also included in the collective evaluation. This perspective might influence the data on the one hand, creating a risk of proxy bias. On the other hand, we saw no other way to give a voice to severely affected PwPPA in our study than to involve those who know them best.

Another limitation is that the effectiveness of *Cope PPA* may have been influenced by many parameters that we cannot adequately consider in the present analysis. For example, no variant-specific differences in terms of the experience of the intervention could be derived from the qualitative data. Investigating parameters such as PPA variant, severity, and depression should be taken into account in future work using statistical models.

By our data-driven analysis, it was not possible to conclusively distinguish between specific and non-specific factors of our intervention. Since the biographic-narrative approach defines very clear therapist behaviour (through predetermined questions, active listening, supported communication), the method cannot be completely separated from the therapeutic relationship. Life story work is always relationship work and, in the authors’ view, has the potential to be used in this sense as well, namely, to get to know people deeply, as Volkmer et al. (2023, [58]) suggest.

## 5. Conclusions

The analysis shows that PwPPA and their family members experienced their participation positively. Not all participants reported changes or improvements regarding their self-image. Some PwPPA had maintained a positive self-image, while others continued to see themselves as deficient. We assume that the mechanisms of action were more likely to result in short-term improvements in mood, which may not have had time to manifest more profoundly due to cognitive deficits. Future studies should examine identity work with PwPPA more closely at an individual level, considering aspects such as resilience as an influencing factor, to determine who would benefit most from biographic-narrative therapy.

## Figures and Tables

**Table 1:** Demographics of PwPPA. ^1^ Age of PwPPA at the start of the study: group 1 and 2—8 April 2024, group 3 and 4—28 April 2025.

Groups	PwPPA (*n*)	Partner/Other (*n*)	Age (Mean, SD) ^1^	*Age at Onset* (*Mean, SD*)	*Age at Diagnosis* (*Mean, SD*)	Sex (F/M, *n*)	Variant (nfvPPA/svPPA/lvPPA/Mixed PPA, *n*)	Pathology (AD/FTLD)	MMSE (Mean, SD)	ST (Mean %)
1	6	4/2	78.0 (7.2)	71.0 (9.1)	74.3 (8.9)	3/3	1/2/3/0	3/3	21.0 (7.1)	88.3%
2	5	5/0	70.0 (10.4)	66.2 (11.1)	69.6 (10.2)	3/2	2/0/2/1	4/1	21.8 (6.0)	78.3%
3	3	2/1	61.0 (12.8)	54.0 (12.8)	60.7 (12.9)	2/1	1/1/1/0	1/2	19.3 (7.4)	97.2%
4	3	3/1	71.3 (7.5)	67.7 (7.5)	70.0 (6.1)	0/3	0/2/0/1	3/0	21.3 (2.1)	96.8%

^1^ PwPPA = people with primary progressive aphasia; F = female; M = male; nfvPPA = nonfluent variant of PPA; svPPA = semantic variant of PPA; lvPPA = logopenic variant of PPA; mixed = mixed variant of PPA; AD = Alzheimer’s disease; FTLD = frontotemporal lobar degeneration.

## Data Availability

The data are not publicly available due to privacy and ethical restrictions.

## Supplementary Materials

The following supporting information can be downloaded at: https://www.mdpi.com/article/10.3390/brainsci16020233/s1, Table S1: SRQR Checklist; Table S2: Overview of the Cope PPA Manual; Table S3: Interview Guidelines; Table S4: Braun & Clarke 15-Point Thematic Analysis Checklist.

## Abbreviations

The following abbreviations are used in this manuscript:

AD	Alzheimer’s disease
FTLD	Frontotemporal lobar degeneration
lvPPA	Logopenic variant of PPA
MADRS	Montgomery–Åsberg-Depression Rating Scale
MMSE	Mini-Mental State Examination
nfvPPA	Nonfluent variant of PPA
PPA	Primary Progressive Aphasia
PwPPA	People with PPA
QoL	Quality of Life
ST	Scenario Test
svPPA	Semantic variant of PPA
