# Supplementary material for: ‘I Am No Longer Anxious When I Speak’: Experiences of People with Primary Progressive Aphasia Taking Part in a Biographic-Narrative Therapy (Cope PPA)
Source: Brain Sci. 2026 Feb 16;16(2):233. doi: 10.3390/brainsci16020233 (PMC12939136; doi:10.3390/brainsci16020233)
Supplement: Supplementary file 1 [file brainsci-16-00233-s001.zip › brainsci-4029877-supplementary.pdf]

Table S1: Standards for Reporting Qualitative Research (SRQR)[a]

N°	Topic	Item	Page	Check
**Title and abstract**				
S1	Title	Concise description of the nature and topic of the study Identifying the study as qualitative or indicating the approach (e.g., ethnography, grounded theory) or data collection methods (e.g., interview, focus group) is recommended	1	✓
S2	Abstract	Summary of key elements of the study using the abstract format of the intended publication; typically includes background, purpose, methods, results, and conclusions	1	✓
**Introduction**				
S3	Problem formulation	Description and significance of the problem/phenomenon studied; review of relevant theory and empirical work; problem statement	1-3	✓
S4	Purpose or research question	Purpose of the study and specific objectives or questions	3	✓
**Methods**				
S5	Qualitative approach and research paradigm	Qualitative approach (e.g., ethnography, grounded theory, case study, phenomenology, narrative research) and guiding theory if appropriate; identifying the research paradigm (e.g., postpositivist, constructivist/interpretivist) is also recommended; rationale[b]	6	✓
S6	Researcher characteristics and reflexivity	Researchers' characteristics that may influence the research, including personal attributes, qualifications/experience, relationship with participants, assumptions, and/or presuppositions; potential or actual interaction between researchers' characteristics and the research questions, approach, methods, results, and/or transferability	3	✓
S7	Context	Setting/site and salient contextual factors; rationale[b]	3	✓
S8	Sampling strategy	How and why research participants, documents, or events were selected; criteria for deciding when no further sampling was necessary (e.g., sampling saturation); rationale[b]	3-4	✓
S9	Ethical issues pertaining to human subjects	Documentation of approval by an appropriate ethics review board and participant consent, or explanation for lack thereof; other confidentiality and data security issues	3	✓
S10	Data collection methods	Types of data collected; details of data collection procedures including (as appropriate) start and stop dates of data collection and analysis, iterative process, triangulation of sources/methods, and modification of procedures in response to evolving study findings; rationale[b]	5-6	✓
S11	Data collection instruments and technologies	Description of instruments (e.g., interview guides, questionnaires) and devices (e.g., audio recorders) used for data collection; if/how the instrument(s) changed over the course of the study	5-6	✓
S12	Units of study	Number and relevant characteristics of participants, documents, or events included in the study; level of participation (could be reported in results)	7-8	✓
S13	Data processing	Methods for processing data prior to and during analysis, including transcription, data entry, data management and security, verification of data integrity, data coding, and anonymization/deidentification of excerpts	6	✓

S14	Data analysis	Process by which inferences, themes, etc., were identified and developed, including the researchers involved in data analysis; usually references a specific paradigm or approach; rationale[b]	6-7	✓
S15	Techniques to enhance trustworthiness	Techniques to enhance trustworthiness and credibility of data analysis (e.g., member checking, audit trail, triangulation); rationale[b]	6	✓
**Results/findings**				
S16	Synthesis and interpretation	Main findings (e.g., interpretations, inferences, and themes); might include development of a theory or model, or integration with prior research or theory	7-12	✓
S17	Links to empirical data	Evidence (e.g., quotes, field notes, text excerpts, photographs) to substantiate analytic findings	7-12	✓
**Discussion**				
S18	Integration with prior work, implications, transferability, and contribution(s) to the field	Short summary of main findings; explanation of how findings and conclusions connect to, support, elaborate on, or challenge conclusions of earlier scholarship; discussion of scope of application/generalizability; identification of unique contribution(s) to scholarship in a discipline or field	12-14	✓
S19	Limitations	Trustworthiness and limitations of findings	14	✓
**Other**				
S20	Conflicts of interest	Potential sources of influence or perceived influence on study conduct and conclusions; how these were managed	15	✓
S21	Funding	Sources of funding and other support; role of funders in data collection, interpretation, and reporting	15	✓

[a]The authors created the SRQR by searching the literature to identify guidelines, reporting standards, and critical appraisal criteria for qualitative research; reviewing the reference lists of retrieved sources; and contacting experts to gain feedback. The SRQR aims to improve the transparency of all aspects of qualitative research by providing clear standards for reporting qualitative research.

[b]The rationale should briefly discuss the justification for choosing that theory, approach, method, or technique rather than other options available, the assumptions and limitations implicit in those choices, and how those choices influence study conclusions and transferability. As appropriate, the rationale for several items might be discussed together.

Table S2: Overview of the *Cope PPA* manual

**Week 1**	**First individual therapy (90 min.)**<br>- VAS scale (before / after)<br>- Shared decision making<br>- Decision on a life story product<br>- Initial narrative question (*'Please tell me your life story. All the events which were important for you. Start wherever you like. Take the time you need. I'll listen first. I won't interrupt. I'll just take some notes.'*)<br>- Feedback<br><br><u>Material</u>: *Cope PPA* manual; document 'Shared Decision Making', pictograms; pen & paper; template 'life cake' / 'mind map'; life story book / memory box; VAS scale; feedback cards	
**Week 2**	**Second individual therapy (90 min.)**<br>- Working on the life story product<br>- Continuation of the main narrative	**First group therapy (90 min.)**<br>**'Get to know each other'**<br>- Introduction with tangible objects

	- <u>Narrative-pointed, internal enquiries</u> (e.g. *'You mentioned your childhood. What else comes to your mind about that time?'*)<br>- <u>Narrative-pointed, external enquiries</u> (e.g. *'Who or what makes you happy?'*)<br>- Preparation of first group therapy<br>- Feedback<br><br><u>Material</u>: *Cope PPA* manual; pictograms; pen & paper; template 'life cake' / 'mind map'; life story book / memory box; feedback cards; participants' photographs	- Communication rules<br>- What is 'quality of life'?<br>- Shared decision making<br>- Feedback<br><br><u>Material</u>: Flipchart with communication rules and goals of *Cope PPA*; prepared word cards with topics of group sessions/expectations; empty word cards to note personal definitions of QoL; VAS scale
**Week 3**	**Third individual therapy (90 min.)**<br>- Working on the life story product<br>- Continuation of the main narrative<br>- Narrative-pointed, internal enquiries<br>- Narrative-pointed, external enquiries<br>- Preparation of second/third group therapy (e.g. timeline and newspaper / magazines)<br>- Feedback<br><br><u>Material</u>: (see Week 2)	**Second group therapy (90 min.)**<br>**'Life course'**<br>- Storytelling using photographs / timelines<br>- Feedback<br><br><u>Material</u>: Wooden board; A3 drawing paper; paintbrushes; gouache; laptop; participants' photographs; cards from art therapy
**Week 4**		**Third group therapy (90 min.)**<br>**'Family / friends'**<br>- Storytelling<br>- Feedback<br><br><u>Material</u>: ball of wool; participants' photographs; white stones; acrylic pens
**Week 5**	**Break**	
**Week 6**	**Fourth individual therapy (90 min.)**<br>**'Hobbies / leisure activities' and 'Current events'**<br>- Working on the life story product<br>- <u>Questions aimed at semantic knowledge</u> (e.g. *'What does health mean to you?'*)<br>- <u>Questions aimed at episodic knowledge</u> (e.g. *'Has your understanding of health changed after the diagnosis?'*)<br>- Preparation of fourth/fifth group therapy (e.g. selection of a newspaper article)<br>- Feedback<br><br><u>Material:</u> (see Week 2)	**Fourth group therapy (90 min.)**<br>**'Hobbies / leisure activities'**<br>- Storytelling<br>- Discussion about photographs and formative experiences<br>- Feedback<br><br><u>Material</u>: tangible objects brought along
**Week 7**		**Fifth group therapy (90 min.)**<br>**'Current events'**<br>- Explaining the procedure<br>- Presentation daily schedule<br>- Storytelling<br>- Feedback<br><br><u>Material:</u> newspaper / magazines; Lego
**Week 8**	**Break**	
**Week 9**	**Fifth individual therapy (90 min.)**<br>**‚Health disease' and 'Job / retirement'**	**Sixth group therapy (90 min.)**<br>**'Health / disease'' or 'Job / retirement'**

	- Working on the life story product<br>- <u>Questions aimed at semantic knowledge</u> (e.g. *'What does health mean to you?'*)<br>- <u>Questions aimed at episodic knowledge</u> (e.g. *'Has your understanding of health changed after the stroke?'*)<br>- Reflecting expectations / Feedback<br><br><u>Material:</u> (see Week 1)	- Explaning procedure<br>- Storytelling<br>- Feedback<br><br><u>Material:</u> Picture cards with health quotes
**Week 10**		**Seventh group therapy (90 min.)**<br>**'Closing / final meeting'**<br>- Explaning procedure<br>- Storytelling<br>- Feedback<br><br><u>Material:</u> (see Week 2)

Table S3: Interview Guide

a) for PwPPA

N°	Question	Additional questions /<br>Memory and communication aids
0	You took part in our study involving individual and group therapy sessions. I would like to find out how you experienced your participation?	-
1	First of all, I would be interested to know: How did you find the therapy? /<br>What was particularly helpful?	How did you find the individual and group therapy sessions?<br><br>What did you think of…<br>- Communication rules<br>- Shared decision making<br>- Topics<br>- Materials<br>- Non-verbal methods (e.g. Lego Serious Play / Painting)<br>- Life Story book<br>- Memory box<br>- Feedback<br>- Exchange with others<br><br>Using photographs of communication rules; timelines; life story books/memory boxes; feedbackcards; etc.<br><br>How did you find the time frame? (Duration/frequency)
2	Did the therapy meet your expectations?	At the beginning of therapy, you said that you expected …<br><br>Using the therapist's notes from the first therapy session concerning shared decision-making<br><br>Have these expectations been fulfilled?

		If yes: To what extent have your expectations been fulfilled?<br><br>*If no:* To what extent have your expectations not been fulfilled?
3	How did you feel during the therapy?	Most of the time, you felt:<br>- Happy<br>- Satisfied<br>- Neutral<br>- Dissatisfied<br>- Unhappy<br><br>Supported by visualisation using the VAS scale
4	What did you gain from the therapy?	Was there anything that helped you in particular?<br><br>Do you now approach conversations with others differently?<br><br>To what extent has the way you deal with your illness changed?
5	How do you see yourself today when you talk to others?	Would you say you see yourself differently since participating in *Cope PPA*?<br><br>Has participating affected your view of:<br>- Your past?<br>- Your life?<br>- Your future?<br><br>*If yes:* in what way?
6	Has anything changed since you took part?	Did participation have an impact on your<br>- family life<br>- social contacts<br>- activities<br>- communication skills?
7	What are you looking forward to when you think about the future?	Do you have anything special planned?
8	Is there anything you would like from us as a research team, or anything you would like to tell us?	*If not*: If you can think of anything else we have overlooked or you would like to add, you can contact us at any time on the number on your flyer (06131-17 2474).<br><br>The next appointment is listed on your flyer. We will also send you a reminder.

b) for family members

N°	Question	Additional questions / Use of examples and materials
0	Your (partner/father-in-law/etc.) took part in our study	-

	involving individual and group therapy sessions. I would like to find out how you felt about the experience.	
1	How do you think your partner experienced the therapy?	How do you think he/she experienced individual and group therapy? How can you tell? Can you give an example?<br><br>As a relative, how did you experience the time frame? (Duration/frequency)<br><br>Using photographs from therapy/ review of life story products
2	You surely had certain expectations at the beginning of the study.<br><br>What were those expectations?	Would you say that these expectations were met?<br><br>*If yes:* To what extent were your expectations met?<br><br>*If no:* To what extent were your expectations not met?
3	What do you think your partner was able to take with them?	Was there anything that particularly helped him/her?<br><br>*If yes:* what?<br><br>Do you think (your husband/wife/sister/mother-in-law) sees himself/herself differently today than before participating?<br><br>*If yes:* what do you think has changed?
4	How was your partner's mood during therapy?	When you look at this scale, what do you think your partner's mood was like? /<br><br>Did his/ her mood change over time?<br>- Happy<br>- Satisfied<br>- Neutral<br>- Dissatisfied<br>- Unhappy<br><br>Supported by visualisation using the VAS scale
5	Has anything changed for you as a family member?	*If yes:* what has changed?
6	What are you looking forward to when you think about the future?	Do you have anything special planned?
7	Is there anything you would like from us as the research team, or anything you would like to tell us?	*If not:* If you can think of anything else we have overlooked or would like to add, you can contact us at any time on the number on your flyer (06131-17 2474).<br><br>The next appointment is listed on the flyer. We will also send you a reminder.

Table S4: Braun & Clarke 15-point Thematic Analysis Checklist[a]

Process	N°	Item	Response
Transcription	1	The data have been transcribed to an appropriate level of detail, and the transcripts have been checked against the tapes for 'accuracy'	Yes. The data have been transcribed orthographically, following the guidelines of Dresing & Pehl (2018). Independent SLT students helped with transcriptions and reviewed transcripts.
Coding	2	Each data item has been given equal attention in the coding process	Yes. During 'Step 1' of our reflexive thematic analysis MG worked through all interviews with the same attention. The coding ('Step 2') was conducted in a systematic way.
	3	Themes have not been generated from a few vivid examples (an anecdotal approach), but instead the coding process has been thorough, inclusive and comprehensive	Yes. The themes have been generated after the coding when all codes were sorted in the Mural Board. The combination of MAXQDA and the Mural Board enabled us to maintain an overview and describe the process as comprehensive today.
	4	All relevant extracts for all each theme have been collated	Yes. We have attempted to summarise the codes belonging to a theme in an understandable way. Our goal was to capture the meaning of the data. The most important excerpts are supported by quotations.
	5	Themes have been checked against each other and back to the original data set	Yes. We heard opinions on the themes through our independent SLT colloquium and also adapted the descriptions based on feedback from the research group (co-authors). In collaboration with JT, the themes have been checked against each other and against the background of the codes.
	6	Themes are internally coherent, consistent, and distinctive	Yes. We have tried to maintain a similar structure and formulate the themes clearly.
Analysis	7	Data have been analysed-interpreted,	Yes. The collaboration between MG and JT helped us achieve the necessary level of

		made sense of- rather than just paraphrased or described	reflection. Discussions within the research team and in the colloquium also helped us to tell a story through the themes.
	8	Analysis and data match each other- the extracts illustrate the analytic claims	Yes. By following Braun & Clarke's step-by-step approach, we feel that the analysis results match the data very well.
	9	Analysis tells a convincing and wellorganised story about the data and topic	Yes. We find this story convincing and well-organised and describe this in our discussion as well.
	10	A good balance between analytical narrative and illustrative extracts is provided	Yes. We tried to maintain a good balance between illustrative and analytical storytelling. It was important to us not to paraphrase the data, but to explain what the data tells us.
Overall	11	Enough time has been allocated to complete all phases of the analysis adequately, without rushing a phase or giving it a once-over-lightly	Yes. We took our time with each step. No phases were skipped. If it seemed that the necessary level of reflection had not yet been reached, we returned to a previous phase.
Written report	12	The assumptions about, and specific approach to, thematic analysis are clearly explicated	Yes. We have made an effort to establish our methodological position and have aligned ourselves with the "constructionist approach" (p. 6). Aspects such as language and meaningfulness in the data have become important to us.
	13	There is good fit between what you claim you do, and what you show you have done- i.e. described method and reported analysis are consistent	Yes. There is good agreement between the described method and the reported analysis.
	14	The language and concepts used in the report are consistent with the epistemological position of the analysis	Yes. The language aligns with our methodological approach.
	15	The researcher is positioned as active in	Yes. The first author either carried out all relevant steps of

		the research process; themes do not just ‘emerge’	data collection and analysis herself or closely supervised them.

[a]Braun V, Clarke V. Successful qualitative research: a practical guide for beginners. London: SAGE Publications Ltd; 2013.
